# Adsorption of Polymer-Tethered Particles on Solid Surfaces

**DOI:** 10.1021/acs.jpcb.1c10418

**Published:** 2022-02-03

**Authors:** Tomasz Staszewski, Małgorzata Borówko, Patrycja Boguta

**Affiliations:** † Department of Theoretical Chemistry, Institute of Chemical Sciences, Faculty of Chemistry, Maria Curie-Skłodowska University in Lublin, 20-031 Lublin, Poland; ‡ Institute of Agrophysics, Polish Academy of Sciences, Doświadczalna 4, 20-290 Lublin, Poland

## Abstract

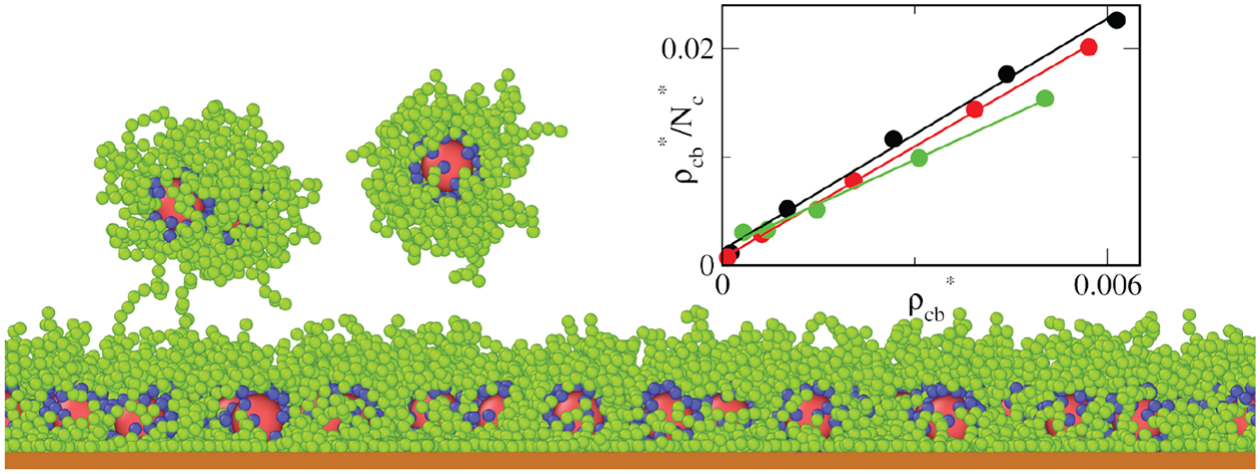

We explore the behavior of polymer-tethered particles on solid surfaces using coarse-grained molecular dynamics simulations. Segment–segment, segment–core, and core–core interactions are assumed to be purely repulsive, while the segment–substrate interactions are attractive. We analyze changes in the internal structure of single hairy particles on the surfaces with the increasing strength of the segment–substrate interactions. For this purpose, we calculate the density profiles along the *x*, *y*, *z* axes and the mass dipole moments. The adsorbed hairy particles are found to be symmetrical in a plane parallel to the substrate but strongly asymmetric in the vertical direction. On stronger adsorbents, the particle canopies become flattened and the cores lie closer to the wall. We consider the adsorption of hairy nanoparticles dispersed in systems of different initial particle densities. We show how the strength of segment–substrate interactions affects the structure of the adsorbed phase, the particle–wall potential of the average force, the excess adsorption isotherms, and the real adsorption isotherms.

## Introduction

Inorganic nanoparticles modified with polymeric ligands, called “hairy” particles, naturally combine the unique physical features of both cores and organic coatings.^1,2^ Such nanoparticles have recently become a focus of materials research since they can be used in a variety of applications such as electronics^3^ optics,^4^ the production of nanocomposites,^5^ biotechnology, and biomedicine.^6−8^ In particular, gold and silver nanoparticles functionalized by ligands of biological importance show great potential for medical progress.^9,10^

The properties of functionalized nanoparticles can be flexibly tuned by varying the grafting density, the length of chains, and the size of the core. Moreover, their solvation and electrochemical properties can be changed easily by modifying the terminal groups from more hydrophilic groups to hydrophobic ones.^11^

Regardless of practical motivations for investigating polymer-tethered nanoparticles, these systems are extremely interesting from a cognitive point of view. Therefore, the behavior of ligand-tethered particles in different bulk systems has been extensively investigated using both experimental and theoretical methods.^1,12^ Most of the research concentrated on modeling the morphology of polymer coatings by changing ligand properties, the grafting density, the interactions of chains with the environment, and the temperature. Several works used the similarity between the polymer-tethered particles and star polymers. Ohno et al.^13,14^ extended the mean-field theory of star polymers^15^ to the polymer-tethered spherical particles of different sizes. The self-consistent field model and the scaling theory were also used to study configurations of chains tethered on spherical particles.^16,17^ The effects of the particle size and the solvent quality on the polymer layer structure were analyzed. In turn, Lo Verso et al.^18^ applied density functional theory to study polymers end-grafted to spherical nanoparticles under good solvent conditions. Recently, Ginzburg^19^ used an SCF-DFT approach to show that neat hairy particles form lamellar, cylindrical, and spherical phases for various nanoparticle volume fractions. This issue was widely explored using fully atomistic molecular simulations;^20−27^ Grest’s group^21−24^ studied spherical particles modified with various ligands solvated in water and organic solvents. Chew et al.^25^ explored the hydrophobicity of monolayer-protected gold nanoparticles and showed that the local hydration free energies at the nanoparticle–water interface were correlated with the preferential binding of propane as a representative hydrophobic probe molecule. Giri and Spohr^26,27^ investigated the alkanethiol chain-covered gold nanoparticles in an aqueous NaCl solution. Special attention was given to the penetration depth of water and ions into the diffuse polymer shell and its dependence on grafting density and functionalization. Moreover, charged monolayer-protected gold nanoparticles have been studied in an aqueous solution at physiological temperature.^28^ Particular attention was paid to electrostatic properties that modulate the formation of a complex comprising the nanoparticle together with surrounding ions and water. The reorganization of ligands tethered to nanoparticles under different environmental conditions was also studied.^29,30^ The resulting heterogeneity of the polymer shell can cause the formation of “patchy” nanoparticles. Choueiri et al.^29^ have experimentally demonstrated the formation of various patchy colloids. Staszewski^30^ applied coarse-grained molecular dynamics simulations of the behavior of mobile ligands on the surface of nanospheres and analyzed the influence of the type of ligands, their number, and the strength of interactions on the structure of the polymer coating. Similar simulations were used to study the behavior of polymer-tethered particles immersed in fluids of isotropic particles.^31^ It has been shown that adsorption of isotropic particles “on chains” causes reconfiguration of the tethered chains, leading to a variety of morphologies, including typical core–shell structures and octopus-like and corn-like structures.^31^

Numerous studies have focused on the self-assembly of hairy particles in bulk systems, as these particles are promising building blocks for the production of novel nanocomposites.^5^ The purpose of this research was to understand the mechanism of the self-assembly and mechanical response of these materials.^5,32−37^ Moreover, functionalized nanoparticles are commonly used to stabilize Pickering emulsions. For this reason, a lot of research has been done on the properties of the liquid–liquid interface with hairy particles.^38^

Relatively little attention has been paid to the study of polymer-tethered nanoparticles on solid surfaces.^39−44^ Depositing hairy particles on a substrate changes the grafted layer structure and can allow them to form highly ordered arrays. The structure of the layer formed by polymer-tethered particles at the surface is dependent on the ligand properties, surface chemistry, and quality of solvent or the kind of deposition process used. The interactions between polymer-tethered particles and the substrate affect their wetting–dewetting stability.^39^ Che et al.^40^ studied monolayers of polystyrene-grafted gold nanoparticles adsorbed on different surfaces. They showed that, with increasing polymer–surface interaction energy, the polymer “canopy” of individual particles spreads out to increase its interaction with the surface. Their submonolayer films contained strings of particles, whereas the monolayer consisted of well-ordered hexagonally arranged particles. These findings are supported by molecular dynamics simulation carried out in refs (41 and 42). The mechanism of adsorption of monotethered nanoparticles on solid surfaces was investigated using computer simulations.^44^ Depending on the assumed parameters, the monotethered particles were adsorbed as single particles or as different aggregates, and the morphology of the adsorbed layer depended mainly on the type of the surface.^44^

Another important issue is to understand the interactions of functionalized nanoparticles with the biological environment.^9,10^ Interactions with lipid membranes affect the corona morphology of nanoparticles as the particles pass through pathways in vivo or experience biological fluids during in vitro applications. Despite significant recent advancements in experimental techniques, a comprehensive understanding of the structure and dynamics of hairy nanoparticles at biointerfaces is still lacking.

The increasing number of various commercial products containing nanoparticles create a new type of nanowaste.^45−48^ These materials can release nanoparticles into the environment. There is heightened concern about the long-term negative effects of nanoparticles due to their potential toxicity.^45^ For example, silver nanoparticles are a leader in the fight against the pathogenic microbial activity. On the other hand, however, it can be perceived as an eco-toxic hazard or as a product bioaccumulating in the trophic chain. Therefore, it is necessary to develop methods of removing such pollutants. Several methods are used to remove nanoparticles from water, namely, aeration, coagulation, and adsorption.^45,46^ The aeration is rather complicated and time-consuming, while the coagulation involves the use of toxic materials as coagulants. However, the adsorption-based method seems to be the most effective and safe procedure for the removal of nanoparticles.^45^ Despite its practical importance, the adsorption of nanoparticles on solid surfaces has not been widely studied so far. Nanoparticles are generally investigated as the adsorbent^31,45,46,49^ but not adsorbate. Hence, it is important to develop insight into the mechanism of adsorption of hairy nanoparticles at solid surfaces. There is still a lack of systematic investigation concerning the correlation between the properties of functionalized nanoparticles and the efficiency of their adsorption on substrates and the structure of the surface layer.

In this work, we study an idealized coarse-grained model for the adsorption of polymer-tethered particles on a flat surface using molecular dynamics simulations. Our main goal is to explore the impact of the substrate on the adsorption of hairy particles and the morphology of the adsorbed layer. We also analyze the changes in the polymer canopy of isolated hairy particles on the model surfaces.

We consider nanoparticles with short ligands. Due to savings in computation time, it is possible to scan a wider range of densities of the systems and to determine adsorption isotherms for selected model surfaces. Nevertheless, this allows us to capture basic factors determining the adsorption of hairy particles at solid surfaces.

## Model and Simulation Methodology

We consider hairy particles dispersed in an implicit solvent near solid surfaces. For computational efficiency, we coarse-grained our system to reduce the number of “atoms” required. A single polymer-tethered nanoparticle was modeled as a spherical core with attached *f* chains. Each chain consists of *M* tangentially jointed spherical segments of identical diameters σ_s_. The core diameter equals σ_c_. The chain connectivity is provided by the harmonic segment–segment potentials
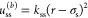
1where *r* is the distance between segments. The first segment of each chain is rigidly fixed to the core at a randomly chosen point on its surface (at the distance σ_cs_ = 0.5(σ_c_ + σ_s_).

All the “atoms” interact via the shifted-force Lennard-Jones potential^50^
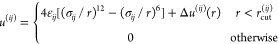
2where
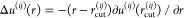
3In eq 3, *r*_cut_^(*ij*)^ is the cutoff distance, σ_*ij*_ = 0.5(σ_*i*_ + σ_*j*_), (*i*, *j* = c, s), and ε_*ij*_ is the parameter characterizing interaction strengths between spherical species *i* and *j*. The indices “c” and “s” correspond to the cores and the chain segments, respectively. To switch on or switch off attractive interactions, we use the cutoff distance. For attractive interactions, *r*_cut_^(*ij*)^ = 2.5σ_*ij*_, while for repulsive interactions, *r*_cut_^(*ij*)^ = σ_*ij*_. Our simulations are based on an implicit solvent model; that is, no solvent molecules are present in the system, but the interactions should be treated as effective, solvent-mediated ones.

The interactions with the substrate are modeled by the potential
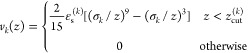
4In eq 4, *z* is the distance from the surface, *z*_cut_^(*k*)^ denotes the cutoff distance, and ε_s_^(*k*)^ is the parameter characterizing interactions of species *k* with the surface (*k* = c, s). To switch on or switch off attractive interactions, we use the cutoff distance parameters, at *v*_*k*_(*z*) = 0.

The standard units are used. The diameter of segments is the distance unit, σ = σ_s_, and the segment–segment energy parameter, ε = ε_ss_ is the energy unit. The mass of a single segment is the mass unity, *m* = *m*_s_. The basic unit of time is . The particles are assumed to be sufficiently lightweight so that the gravity effect is negligible. The energy constants of the binding potentials, *k*_cs_ and *k*_ss_, are 1000ε/σ^2^.

The behavior of the systems depends on the strengths of interactions between all single entities, cores and segments, as well as their interactions with the substrate. Various models were considered for bulk systems involving hairy particles. Lafitte et al.^35^ modeled systems with strong attractive core–core interactions, weak attractive segment–segment interactions, and changed cross-interactions from attractive to nearly purely repulsive. This was used to describe solvent-free silica nanoparticles decorated with organic ligands. However, simpler models were usually used in simulations of hairy nanoparticles. Most of the works assumed purely repulsive cross-interactions to mimic incompatibility between cores and polymers. Among the latter models we can distinguish four classes, namely, those with (i) all repulsive interactions,^51^ (ii) all attractive interactions,^36^ (iii) the attractive core–core interaction and the repulsive segment–segment interactions,^1^ and inversely, (iv) the repulsive core–core interactions and the attractive segment–segment interaction.^37,52,53^ Moreover, different interactions with the surface can be considered: (i) all repulsive interactions, (ii) all attractive interactions, (ii) attractive core–substrate interactions and repulsive segment–substrate interactions, and (iv) attractive segment–substrate interactions and attractive segment–substrate interactions. For surface systems, all combinations of the above models can be considered. In this work, we limited ourselves to analysis of only one type of model systems. We assumed that core–core, core–segment, and segment–segment interactions were purely repulsive. However, core–substrate interactions were repulsive, while segment–substrate interactions were attractive. In the framework of the implicit solvent model, our model corresponds to the good solvent conditions.^18^ In other words, the tethered chains are solvophilic, while the substrate is solvophobic.

In this article, we use standard reduced quantities, reduced distances l* = *l*/σ, and reduced energies E* = *E*/ε. The usual definition of the reduced temperature is introduced, T* = *k*_B_*T*/ε, where *k*_B_ is the Boltzmann constant.

In the remaining part of the work, we use the simpler symbol for the energy parameter ε_s_^*(*s*)^ = ε_s_^*^. We also introduce the reduced densities: the reduced density of cores, ρ_c_^*^σ_c_^3^ = *Nσ*_c_^3^/*V*, the reduced density of segments, ρ_s_^*^ = *NMfσ*_s_^3^/*V*, and the total reduced density, ρ_t_^*^ = ρ_c_^*^ + ρ_s_^*^, where *N* is the number of particles (cores) and *V* is the volume of the system.

We focus our attention on the adsorption of hairy particles. To determine the adsorption isotherms, a very large number of particles in the system is needed. In order to limit the total number of “atoms”, calculations were performed for short ligands. However, in coarse-grained models of polymers, the segments can represent several molecular fragments. Depending on the conditions, each bead can comprise 1–3 (or even more) such the groups.^12^

In our simulations, *M* = 10, *f* = 30, ε_ij_^*^ = 1 (*i*, *j* = c, s), and ε_s_^*(*c*)^ = 1. The mass of the core is arbitrarily set to *m*_c_ = 4*m*_s_. Of course, the dynamic properties of the system would depend on the masses of all species. Our interest, however, is only in the evaluation of the equilibrium structure of the system. We studied particles with different segment–substrate interactions for which ε_s_^*^ = 1, 2, 3, 4, 5, 6. Similar parameters were in the previous simulations of hairy particles.^31,35,43,49^ Calculations were carried out for different initial densities of the particles, ρ_*t*0_^*^ = 0.01, 0.02, 0.03, 0.04, 0.05.

Molecular dynamics simulations were carried out using the LAMMPS package.^54,55^ The Nose–Hoover thermostat was applied to regulate the temperature. The reduced temperature was T* = 1. We considered an ensemble of polymer-tethered nanoparticles in a rectangular box of reduced dimensions equal to *L*_x_^*^, *L*_y_^*^, and *L*_z_^*^ along the axes *x*, *y*, and *z*, respectively. Standard periodic boundary conditions in the *x* and *y* directions were assumed. The walls of the box located at z* = 0 and z* = *L*_z_^*^ mimic adsorbent surfaces. The distance *L*_z_^*^ = 140 was large enough to ensure the existence of a region of uniform fluid at the middle of the box (the bulk phase). *L*_x_^*^ = *L*_y_^*^ ranged from 241 to 540. The simulated systems comprised 338625 “atomic units”.

We equilibrated the system for at least 10^7^ time steps until its total energy reached a constant level, at which it fluctuated around a mean value. The production runs were for at least 10^6^ time steps. At the time, data were saved after every 100 time steps and used for the evaluation of the local densities of cores ρ_c_^*^(z*) and segments, ρ_s_^*^(z*) .

We introduced two identical, space-distant surfaces to achieve better precision of the simulation. All physical quantities were calculated as the average of values estimated for both halves of the simulation box.

Examples of the equilibrium configurations were depicted using the OVITO.^56^

## Results and Discussion

### Single Hairy Nanoparticle at a Solid Surface

We begin with the discussion of the behavior of a single hairy nanoparticle on a solid surface. We aim to show the impact of the strength of attractive interactions between tethered chains and the solid surface on the morphology of the polymer canopy. The surfaces with gradually increasing strength of segment–substrate interactions are considered. Simulations were carried out for the energy parameter ε_s_^*^ = 1, 2, 3, 4, 5, 6. In all cases, the hairy particle was adsorbed on the surface.

**Figure 1:**
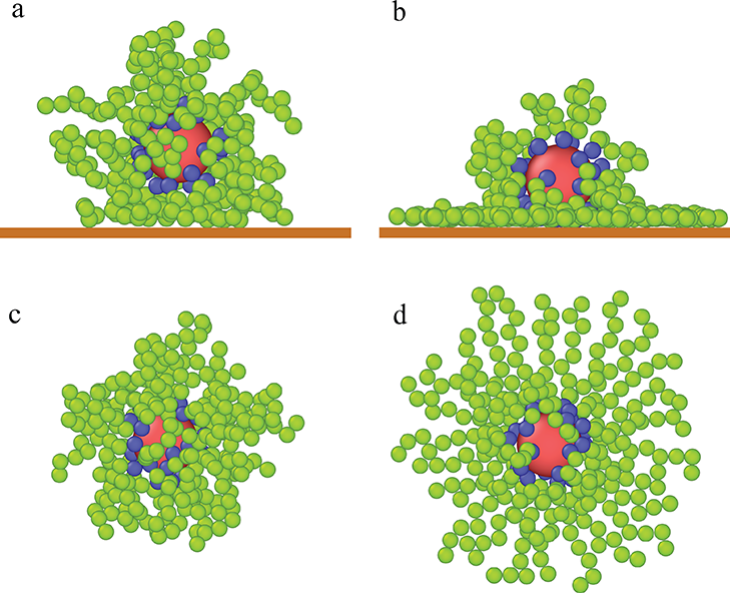
Examples of the equilibrium configurations of hairy particles adsorbed on the surfaces of different strength of segment–substrate interactions, ε_s_^*^ = 1 (a, c) and ε_s_^*^ = 6 (b, d). The red sphere represents the core, blue spheres correspond to bonding segments, and green spheres represent the remaining segments. In parts (a, b), side views of the particles are shown, while parts (c, d) present the views from above.

Figure 1 shows examples of equilibrium configurations of the adsorbed hairy nanoparticles for the lowest value, ε_s_^*^ = 1 (a, c), and the highest value, ε_s_^*^ = 6 (b, d). As expected, increasing strength of attraction causes the chains to spread out on the substrate and the canopy height to decrease (parts a, b). A top view of the adsorbed particles is shown in the lower panel (c, d).

**Figure 2:**
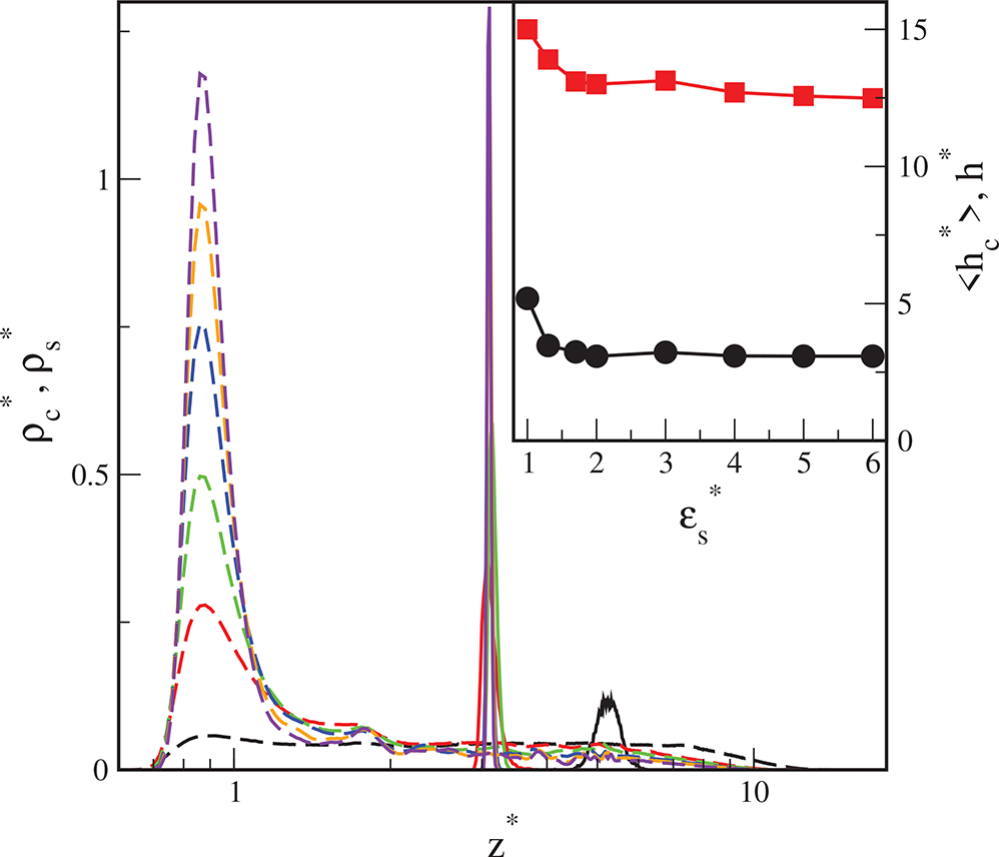
Density profiles of the cores (solid lines) and the chain segments (dashed lines) on the surfaces with different energy parameters ε_s_^*^: 1 (black), 2 (red), 3 (green), 4 (blue), 5 (brown), and 6 (violet). The abscissa is scaled logarithmically. In the inset, the average distance of the core from the substrate, ⟨*h*_c_⟩, (black circles) and “the effective polymer canopy height”, h*, (red squares) are plotted as functions of the energy parameter ε_s_^*^. Symbols correspond to simulation points. Lines serve as a guide to the eye.

Figure 2 depicts the reduced core and segment density profiles for the various ε_s_^*^ parameters. Notice that the reduced densities ρ_c_^*^ and ρ_s_^*^ are proportional to the volume occupied by the cores and segments, respectively. Here we can see that the behavior of the hairy particle on the weak adsorbent surface (ε_s_^*^ = 1, black lines) is fundamentally different from its behavior on the other surfaces. All core density profiles have one well-pronounced peak, and its width decrease as the ε_s_^*^ increases. For stronger segment–substrate interactions, the core profiles are located near the wall, and the average height, ⟨*h*_c_⟩, remains almost constant. On the contrary, for ε_s_^*^ = 1 the core profile is shifted toward longer distances from the surface; this is also reflected by the high value of the height ⟨*h*_c_⟩. In the case of the weakest adsorbent, the segment profile is almost constant for 2 < z* < 7.5 and then gradually decreases. However, for all remaining surfaces, the density profiles exhibit high and narrow peaks at z* = 0.87, very low maxima at z* = 0.87, and then the densities smoothly decrease. It can be said that the core rests on a “segment cushion” which has one dense layer of segments and another much less dense. For stronger segment–surface interactions, the segment density near the surface increases, while the opposite effect is seen far away from the wall. In the inset in Figure 2, we present the dependence of the average distance of the cores from the substrate, ⟨*h*_c_⟩, plotted as a function of the energy parameter ε_s_^*^. Initially, with the increase of the energy parameter ε_s_^*^, the distance of cores from the wall smoothly decreases; however, for sufficiently strong segment–substrate interactions it remains almost constant. We also show here “the effective polymer canopy height”, defined as the distance from the substrate where the segment density becomes zero, h*. The course of a function h*(ε_s_^*^) corresponds to the changes in *h*_c_^*^.

**Figure 3:**
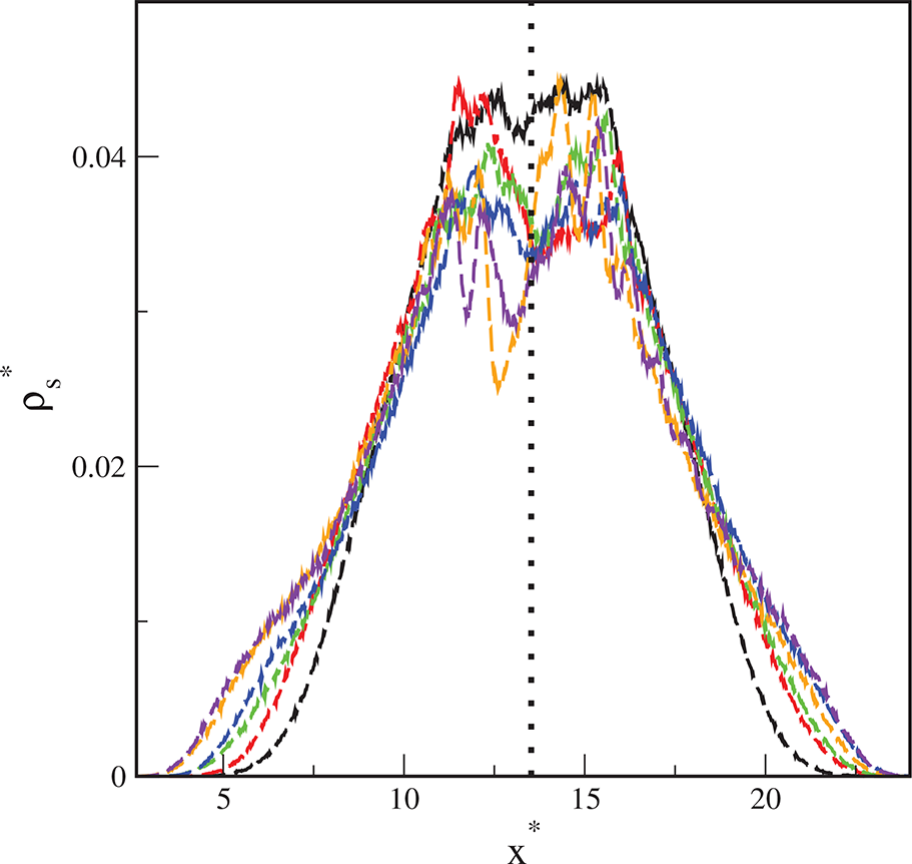
Segment density profiles (dashed lines) along the *x*-axis on the surfaces with different energy parameters ε_s_^*^: 1 (black), 2 (red), 3 (green), 4 (blue), 5 (brown), and 6 (violet). The black, dotted line corresponds to the position of the core.

Next, will focus on the change in the shape of the polymer corona on the surface. Figure 3 shows the average segment density profiles along the *x* direction. The black, dotted line corresponds to the position of the core. In all cases, the symmetrical distributions of the segments are found. As ε_s_^*^ increases, the segment density at greater distances from the core becomes higher. This is also visible in the snapshots from Figure 1c,d. We checked that the profiles ρ*(y*) are almost identical to those of ρ*(x*). Thus, the polymer canopies are symmetrical in the *xy* plane.

**Figure 4:**
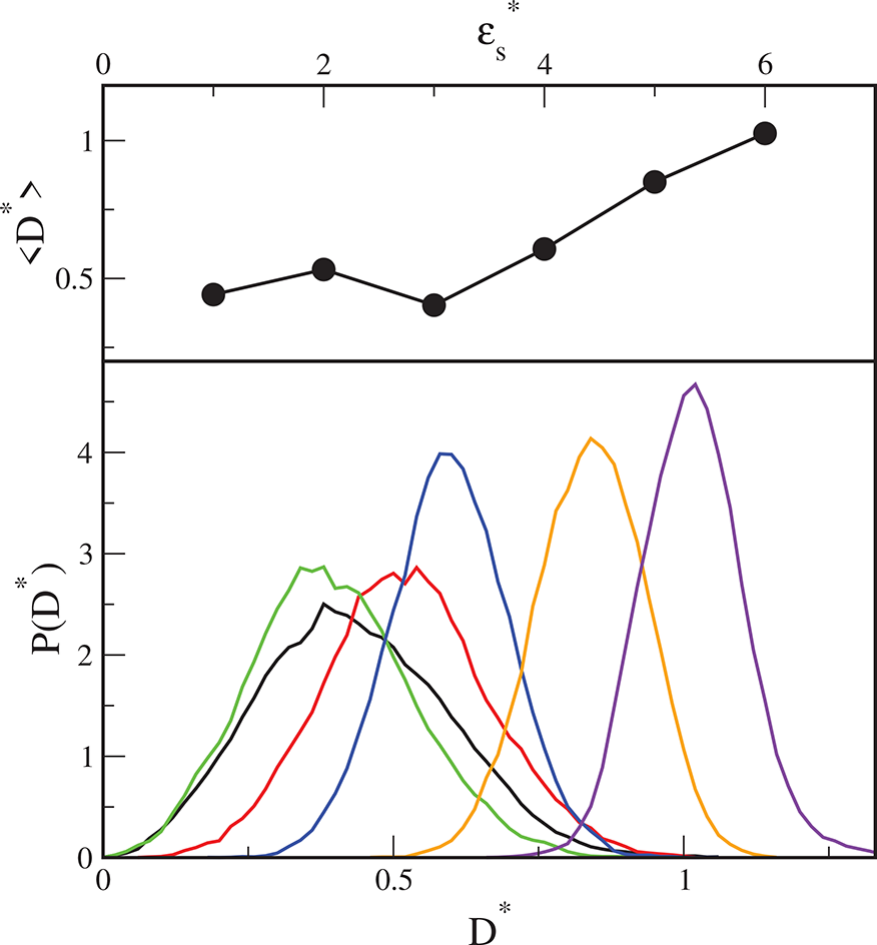
Mass dipole moments of the isolated hairy particle adsorbed on different substrates plotted as a function of the energy parameter ε_s_^*^ (upper panel). Symbols correspond to simulation points. Lines serve as a guide to the eye. Distributions of mass dipole moments for the surfaces with different energy parameters ε_s_^*^: 1 (black), 2 (red), 3 (green), 4 (blue), 5 (brown), and 6 (violet) (bottom panel).

One of the methods for characterizing the distribution of chain segments around the core is based on the concept of the so-called “mass dipole moment”, D*, defined as the distance between the centers of mass of the segments and the core center.^43^ For a perfectly spherical hairy particle, D* = 0, and the increasing value of D* reflects the increasing asymmetry of the polymer canopy. We have studied the polymer coating in the bulk system and found ⟨D*⟩ ≈ 0. The average mass dipole moment as a function of the energy parameter ε_s_^*^ is shown in Figure 4 (upper panel). Below, the corresponding distributions *P*(D*) are plotted. In all cases, ⟨D*⟩ > 0, i.e., the hairy particles become more anisotropic near the substrate. For weak adsorbents, ⟨D*⟩ remains almost constant; however, for stronger interactions with the substrate (ε_s_^*^ > 3), the average mass dipole moment increases. Indeed, on stronger adsorbents, the polymer canopies are more flattened. We monitored the variation of the particle shape over time. The distributions *P*(D*) are plotted in the lower panel of Figure 4. Typical Gaussian histograms *P*(D*) were obtained. The distributions *P*(D*) are broader for lower segment–substrate interactions. This indicates greater variation in the shape of hairy particles on weaker adsorbents. More stable structures are observed for stronger adsorbents.

To understand further the behavior of the adsorbed hairy particles we have also calculated the radius of gyration of the cloud of segments
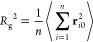
5where **r**_*i*0_ = **r**_*i*_ – **r**_0_, and **r**_*i*_ and **r**_0_ are positions of the *i*th segment and the center of mass, respectively, and *n* = *fM*.

**Figure 5:**
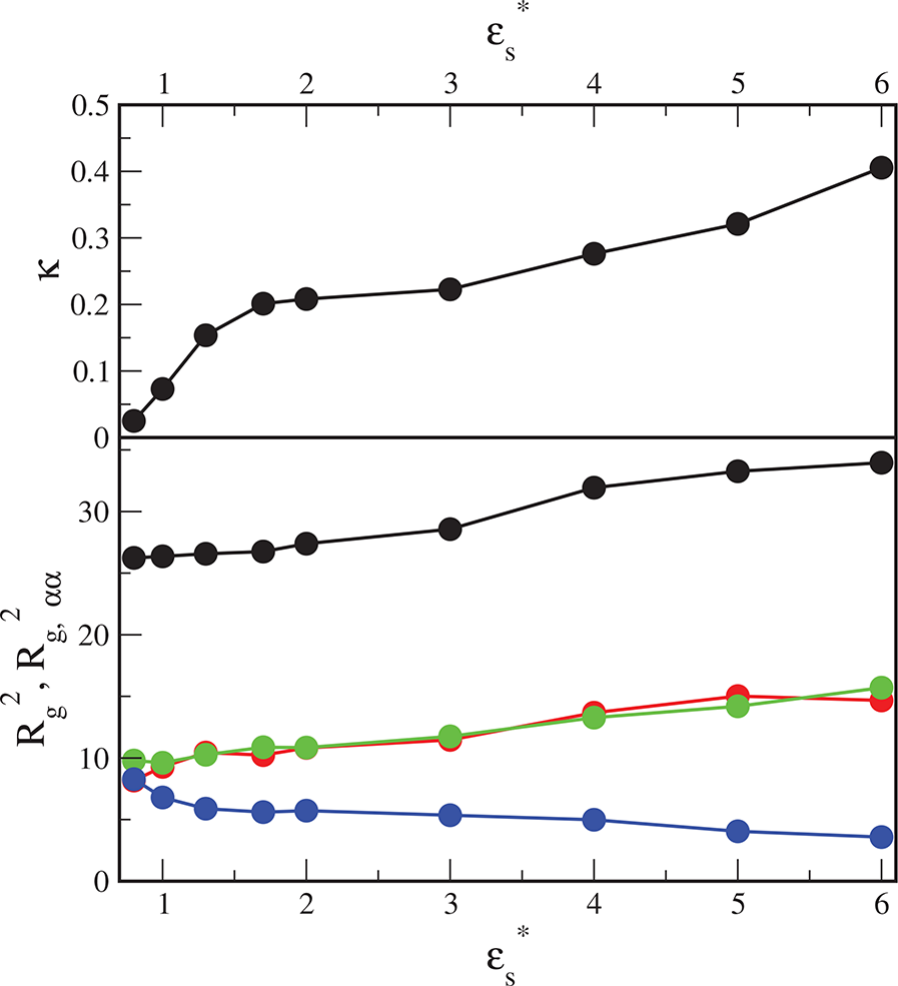
Relative shape anisotropy and the squared radius of gyration and its components *R*_*g*,*xx*_^2^, *R*_*g*,*yy*_^2^, and *R*_*g*,*zz*_^2^ for the isolated particle plotted as functions of the strength of segment–surface interactions.

We resolve the vectors **r**_*i*0_ in eq 5 into components parallel to the axes *x*, *y*, *z* and generate the corresponding radii of gyration labeled *R*_g,*αα*_^2^ (α = *x*, *y*, *z*), the sum of which equals *R*_g_^2^. The results are shown in the low panel of Figure 5. We see here that the squared radius of gyration, *R*_g_^2^, is greater for stronger adsorbents. As expected, the components parellel to the surface are almost the same, *R*_g,*xx*_^2^ = *R*_g,*yy*_^2^, and rise with increasing ε_s_^*^, while the perpendicular component *R*__g,*zz*__^2^ decreases. The adsorbed chains spread laterally on the surface, and the polymer canopy flattens.

To quantify the deformation of the hairy particle near the adsorbing surface, we have additionally determined that the relative shape anisotropy of the segments cloud is defineds as
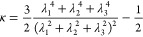
6where λ_1_, λ_2_, and λ_3_ are principal components of the gyration tensor, determined using the procedure described elsewhere.^57^ This parameter is limited between values of 0 and 1. It reaches 1 for a linear chain and drops to zero for spherical conformations. In the case of the considered hairy particles, if segment–substrate interactions become stronger, the relative shape anisotropy of the segment cloud increases, reflecting that it is more asymmetrical (see the top panel in Figure 5).

Our results are consistent with the experimental observations reported by Che et al.^40^ for isolated particles of polystyrene-grafted gold nanoparticles near the substrates with various surface energies. They found that with increasing favorable interactions between the polymer and the substrate, the polymer canopy height decreases as chains spread out to maximize contact with the surface. The previous simulations performed by Ethier and Hall^41^ also confirmed the changes of the polymer coatings near the surface.

### Structure of the Adsorbed Layer

Next, we consider the behavior of the systems with different particle densities. In this case, we consider three model surfaces: S1 (ε_s_^*^ = 1), S2 (ε_s_^*^ = 3), and S3 (ε_s_^*^ = 6). For assumed interaction parameters, the particles accumulate near the surface.

**Figure 6:**
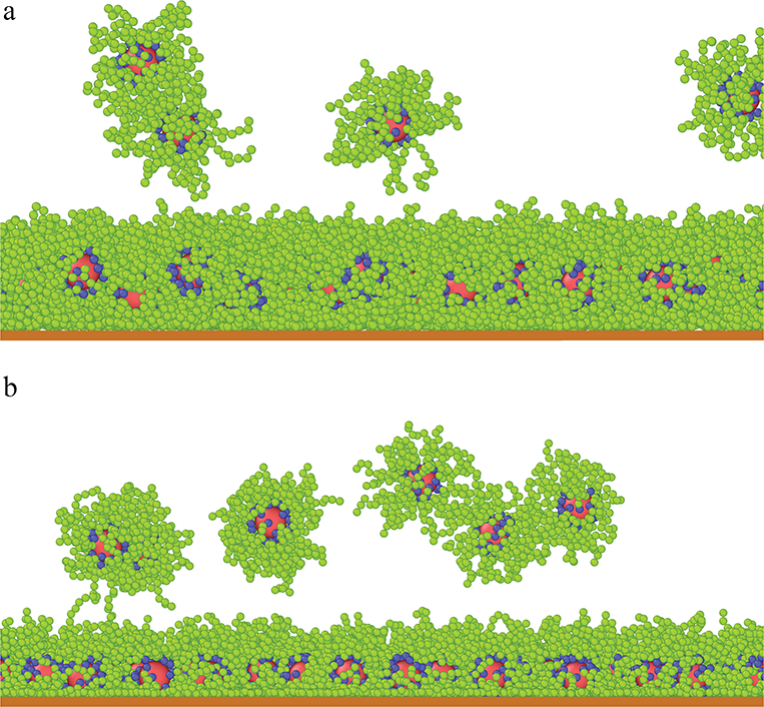
Side views of the equilibrium configurations of hairy particles adsorbed on the surfaces of different strength of segment–substrate interactions, ε_s_^*^ = 1 (a) and ε_s_^*^ = 6 (b). The red sphere represents the core, blue spheres correspond to bonding segments, and green spheres represent the remaining segments. The initial density of the hairy particles, ρ_*t*0_^*^ = 0.01.

We start with the analysis of the structure of the layer formed on the solid surface. Examples of the configurations at the lowest core density are presented in Figure 6. It can be seen that with the increasing strength of chain–substrate interactions, the layer becomes thinner and the cores lie closer to the wall. We have also monitored the configurations of adsorbed chains assuming that such a chain has at least one segment at the surface. The fractions of trains, loops, and tails, respectively, are 0.332, 0.621, and 0.046 for the weak adsorbent (a) and 0.735, 0.256, and 0.009 for the strong adsorbent (b).^58^ As ε_s_^*^ increases, the fraction of trains considerably rises, while fractions of loops and tails decrease.

**Figure 7:**
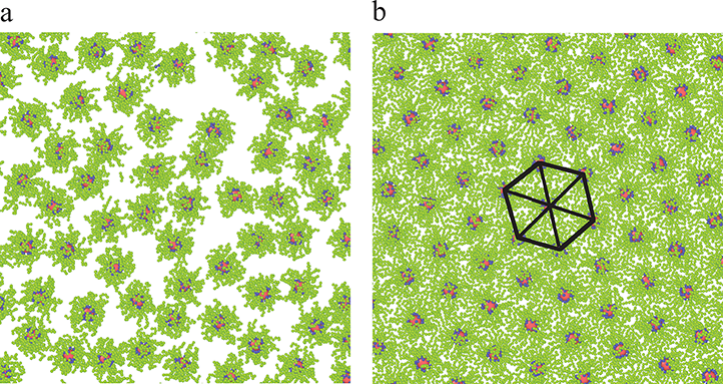
Monolayers of hairy particles formed on the surfaces with different energy parameters, ε_s_^*^ = 1 (a) and ε_s_^*^ = 6 (b) (views from above). The red sphere represents the core, blue spheres correspond to bonding segments, and green spheres represent the remaining segments. The initial density of the hairy particles, ρ_*t*0_^*^ = 0.05.

**Figure 8:**
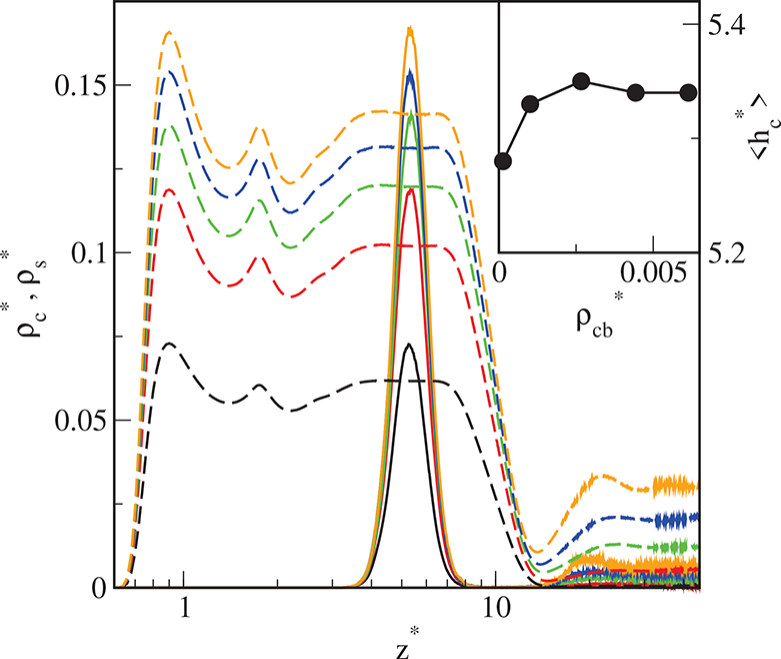
Density profiles of the cores (solid lines) and the chain segments (dashed lines) on the surface S1 for different initial densities of particles in the system ρ_*t*0_^*^: 0.01 (black), 0.02 (red), 0.03 (green), 0.04 (blue), and 0.05 (brown). The abscissa is scaled logarithmically. In the inset, the average distance of the cores from the substrate, ⟨*h*_c_⟩, is plotted as a function of the density of cores in the bulk phase, ρ_cb_^*^. Symbols correspond to simulation points. The line serves as a guide to the eye.

**Figure 9:**
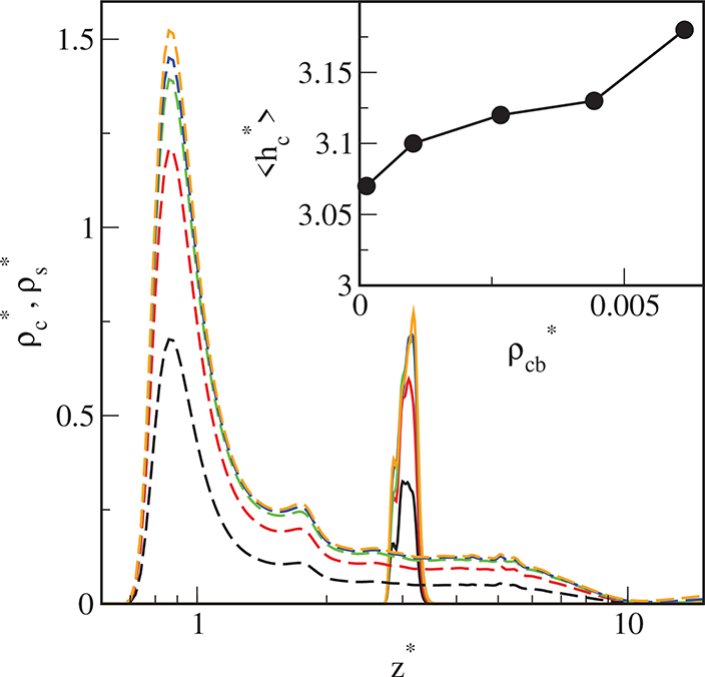
Density profiles of the cores (solid lines) and the chain segments (dashed lines) on the surface S2 for different initial densities of particles in the system ρ_*t*0_^*^: 0.01 (black), 0.02 (red), 0.03 (green), 0.04 (blue), and 0.05 (brown). The abscissa is scaled logarithmically. In the inset, the average distance of the cores from the substrate, ⟨*h*_c_⟩, is plotted as a function of the density of cores in the bulk phase, ρ_cb_^*^. Symbols correspond to simulation points. The line serves as a guide to the eye.

It is interesting to discuss the arrangement of the particles on the surface under different conditions (see Figure 7). In part a, the monolayer for ε_s_^*^ = 1 is shown. In this case, the particles are randomly distributed on the surface. However, for ε_s_^*^ = 6 they form a regular hexagonal lattice (part b). Our results are in line with previous experimental observations. Che el al.^40^ showed that gold nanoparticles modified with polystyrene assembled into rougher, less-ordered monolayers on substrates with low interface energy, while large-area, highly ordered monolayer of these particles can be fabricated only on substrates with high interface energy. In our model, the particle–particle interactions are generally isotropic and repulsive. The particles are energetically inert to each other. Thus, their assembly results only from the entropic effects. We analyzed how the energy parameter ε_s_^*^ and the density of particles in the systems affect the structure of the surface layer. As we will show below, for ε_s_^*^ = 1 and ε_s_^*^ = 3 the density of particles near the surface is quite small (see Figures 7 and 8). In this case, the adsorbed particles remain isolated and can move chaotically on the surface. Such a monolayer is highly disordered. Similar structures were found for ε_s_^*^ = 6 at low densities ρ_*t*0_^*^. However, for a sufficiently dense monolayer (Figure 9) the entropic effects become dominant and the particles assemble into the hexagonal structure that is the optimal packing for spheres in two dimensions. Numerous experiments provided evidence of the formation of hexagonal arrays of hairy particles on surfaces.^1^

**Figure 10:**
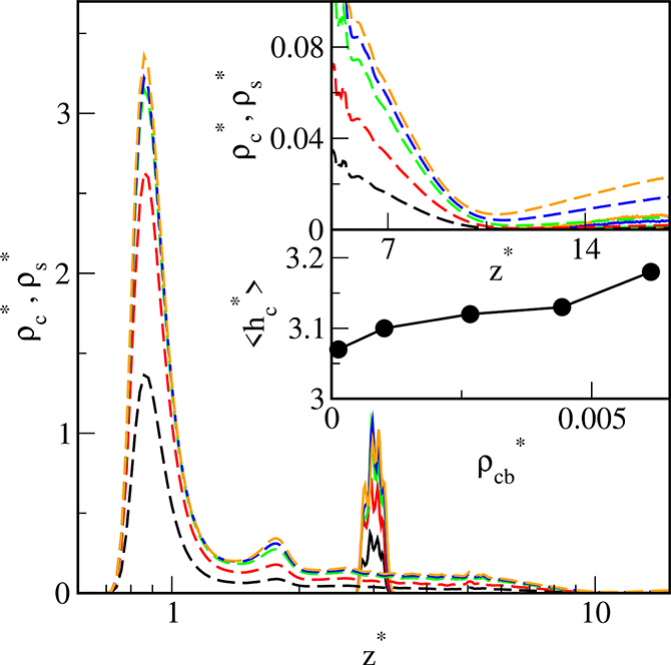
Density profiles of the cores (solid lines) and the chain segments (dashed lines) on the surface S3 for different initial densities of particles in the system ρ_*t*0_^*^: 0.01 (black), 0.02 (red), 0.03 (green), 0.04 (blue), and 0.05 (brown). The abscissa is scaled logarithmically. In the upper panel of the inset, the density profiles of segments in the outer part of the adsorbed layer are drawn on a more precise scale. In the bottom panel, the average distance of the cores from the substrate, ⟨*h*_c_⟩, is plotted as a function of the density of cores in the bulk phase, ρ_cb_^*^. Symbols correspond to simulation points. The line serves as a guide to the eye.

To explore the morphology of the surface film, we calculated the density profiles along the *z*-axis. Figures 8–10 display the reduced density profiles of the cores (solid lines) and the segments (dashed lines) for different initial total densities of the particles, and for three model surfaces, S1 (Figure 7), S2 (Figure 8), and S3 (Figure 9). The structure of the adsorbed layer on the weakly attractive surface S1 differs significantly from that formed on the strong adsorbents S2 and S3.

We can see that all profiles of cores have sharp and high maxima, and their position depends on both the ε_s_^*^ and the density of the particles in the system. The cores are further from the surface S1 than from the substrates S2 and S3. If the bulk density of the cores increases, the average distance of the adsorbed cores ⟨*h*_c_^*^⟩ on the surface S1 initially increases and then reaches a plateau, while for surfaces S2 and S3 it increases monotonically (see insets in Figures 8–10). However, the impact of the core density on ⟨*h*_c_^*^⟩ is not significant.

The reduced density profiles of segments have two maxima near the surface, and in “the core layer” the densities ρ_s_^*^ remain almost constant. The first peaks are well-pronounced and high, while the second ones are markedly lower. Then, the segment density smoothly decreases to very low values. It is noteworthy that these values are lower than the segment densities in the bulk phase. The depletion of segment density at the interface between the bulk phase and the surface film is observed for all considered surfaces (see the inset in Figure 9). This means that there is a repulsive force acting on the particles in the bulk phase. Indeed, the chains tethered to adsorbed cores which are directed toward the bulk phase generate the repulsion. We return to this issue below.

The segment profiles for the particles adsorbed on the S1-substrate differ significantly from the segment profiles for the other two surfaces. For strongly attracting surfaces the peaks at the immediate vicinity of the walls are much higher and narrower, while in the outer part of the canopies, the segment densities are considerably lower.

**Figure 11:**
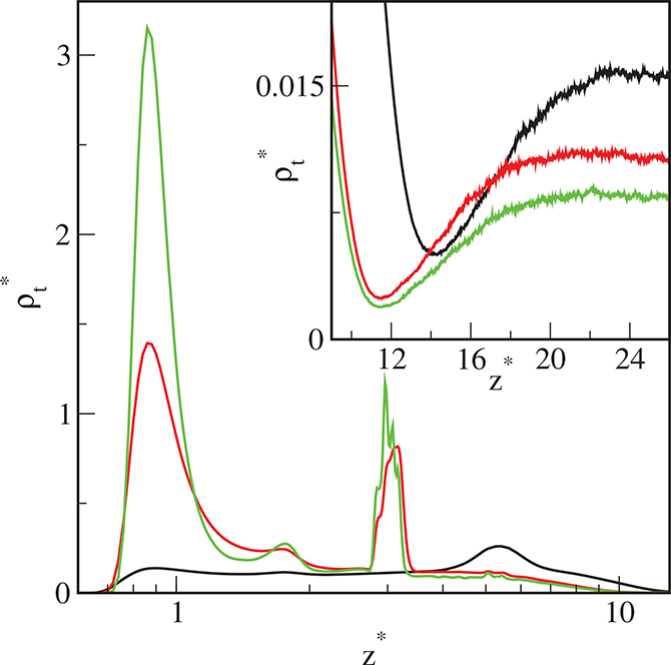
Total density profiles of the hairy nanoparticles on the different surfaces: S1 (black line), S2 (red line), and S3 (green line). The initial total density ρ_*t*0_^*^ = 0.03. The abscissa is scaled logarithmically. In the inset, the total density profiles of particles in the outer part of the adsorbed layer are drawn on a more precise scale.

In Figure 11, the total density profiles, ρ_t_^*^ = ρ_c_^*^ + ρ_s_^*^, are plotted for different surfaces. The presented results were obtained for the moderate initial density of the system ρ_*t*0_^*^ = 0.03. The analysis of these profiles confirms that as the strength of the attractive chain–substrate interaction increases, more and more segments accumulate on the surface and the cores move away from the wall. Notice that the thickness of the adsorbed monolayer decreases as ε_s_^*^ increases. Moreover, there is a depletion in the total fluid density in the outer part of the adsorbed layer (see the inset). The same effect was found for Janus particles adsorbed on solids when the repulsive parts of particles were oriented toward the bulk phase.^59^

To gain deeper insight into the mechanism of adsorption we evaluated the “effective Boltzmann averaged wall-core potentials”, *v*_eff_.^43^ For this purpose, we simulated the density profiles ρ(*z*) at two very low bulk densities ρ_cb_ and extrapolated the ratio ρ(*z*)/ρ_cb_ to zero bulk density. In this manner, we evaluated the Boltzmann function
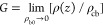
7

The effective core-substrate potential is defined as
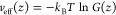
8

**Figure 12:**
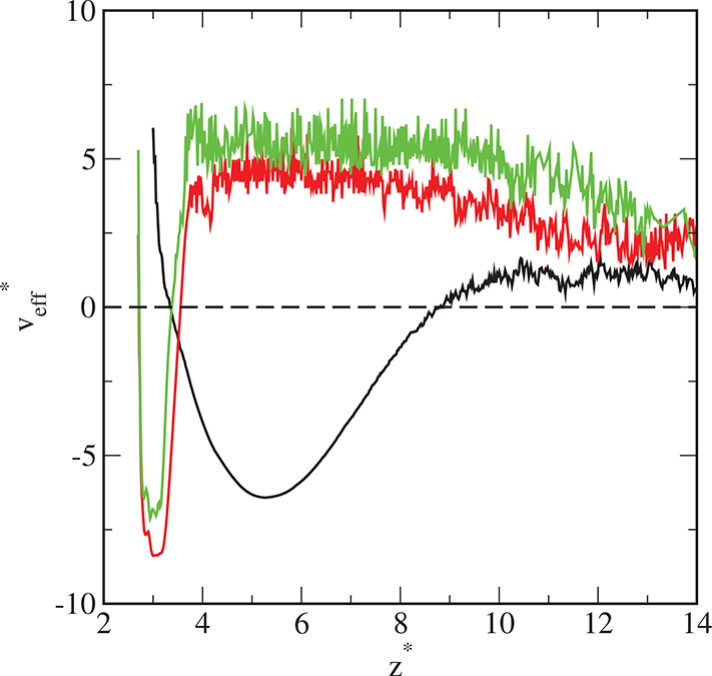
Nanoparticle–wall potential of the average force for the different surfaces: S1 (black line), S2 (red line), and S3 (green line). The initial density of the hairy particles, ρ_*t*0_ = 0.01.

The effective potentials for the surfaces in questions are presented in Figure 12. These potentials exhibit deep attractive wells near the surface and the repulsive regions further from the wall. Obviously, the potentials tend to zero for much greater distances. The attractive well for ε_s_^*^ = 1 is shallower and wider than for greater values of this parameter. It is interesting that the minimum in *v*_eff_^*^ is slightly lower for ε_s_^*^ = 3 than for ε_s_^*^ = 6. For sufficiently strong segment–substrate interactions, a lot of the tethered chains accumulate on the surface and substantially “screen” its attraction. Further from the surface, however, the repulsive parts of effective potentials always increase with increasing ε_s_^*^. The total density profiles (Figure 10) are consistent with the effective potentials, *v*_eff_^*^(z*).

### Adsorption Isotherms

A measure of the adsorption of the particles (cores) is the excess adsorption (per unity of the surface area) are defined as
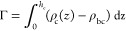
9where ρ_cb_ is the density of the cores in the bulk phase. The latter was determined from the density profile. Sufficiently far away from the substrate, adsorption does not take place and the core density achieves a constant value, ρ_cb_.

The excess adsorption can also be estimated from the following formula
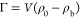
10where *V* is the volume of the adsorption system. The last equation is used for the experimental measurement of adsorption.

**Figure 13:**
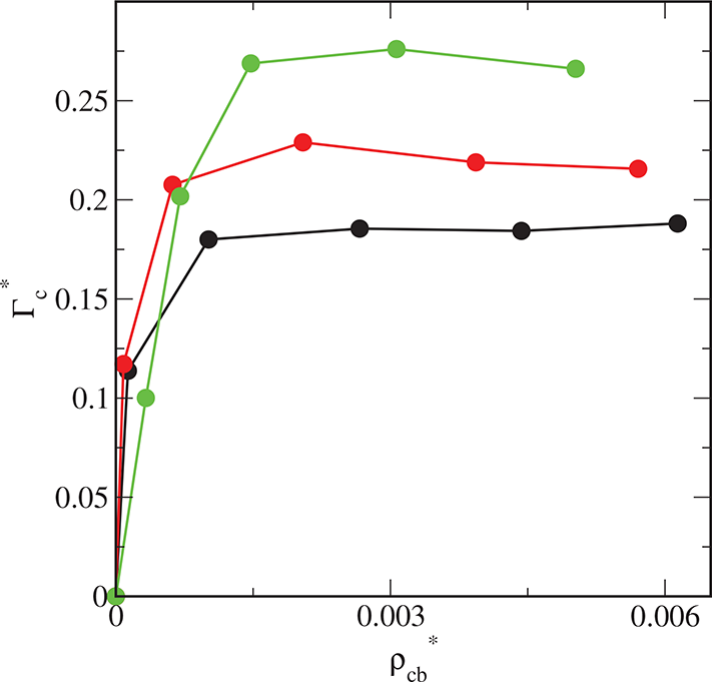
Excess adsorption isotherms of hairy particles on different surfaces: S1 (black), S2 (red), and S3 (green). Γ* = *Γσ*_c_^3^. Symbols correspond to simulation points. Lines serve as a guide to the eye.

In Figure 13, we show the excess adsorption isotherms of nanoparticles (cores) on different solid surfaces (Γ* = *Γσ*_c_^3^). The excess adsorption isotherms have a typical course. When the density ρ_cb_^*^ increases, the excess adsorption rapidly rises, reaches its maximum, and begins to slowly decline. Let us discuss the influence of the energy parameter ε_s_^*^ on the excess adsorption isotherm. In the case of the highest bulk densities considered here, Γ*(ε_s_^*^ = 1) < Γ*(ε_s_^*^ = 3) < Γ*(ε_s_^*^ = 6) . However, for low densities the following relation is found Γ*(ε_s_^*^ = 6) < Γ*(ε_s_^*^ = 1) < Γ*(ε_s_^*^ = 3) .

The real adsorption of particles, meaning the number of cores in the surface film, can be calculated from the equation
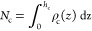
11where *h*_c_ is the thickness of the surface layer. We estimated *h*_c_ from the density profiles of the cores. For low densities in the bulk phase and strong adsorption, Γ ≈ *N*_c_.

**Figure 14:**
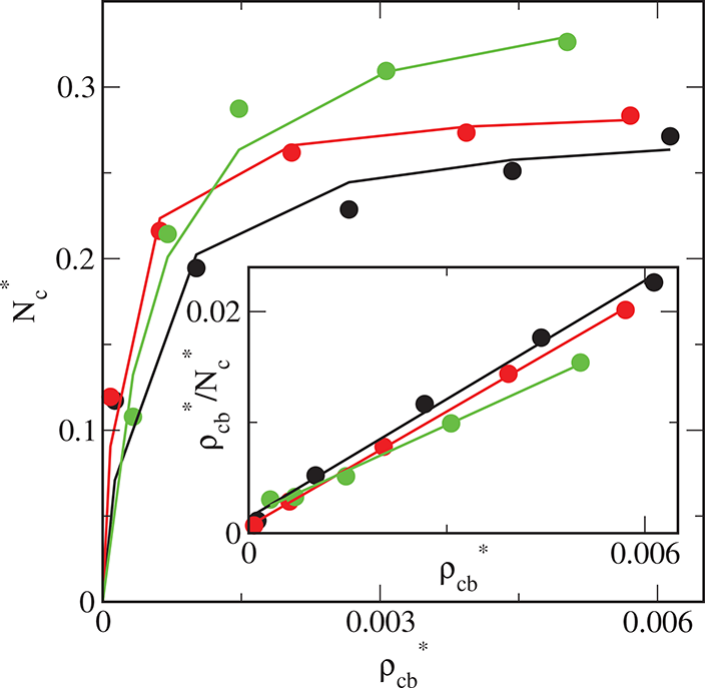
Real adsorption isotherms of hairy particles on different surfaces: S1 (black line), S2 (red line), and S3 (green line) approximated by Langmuir equation (*N*_c_^*^ = *N*_c_σ__c_^3^_). In the inset, Langmuir isotherms are plotted in a linear form. Symbols correspond to simulation points.

We show the real adsorption isotherms obtained from the simulations in Figure 14 (circles). The real adsorption isotherms monotonically increase as the bulk density increases. For high bulk densities, the impact of the parameter ε_s_^*^ on the real adsorption is the same as in the case of the excess adsorption isotherms.

Adsorption isotherms can be calculated from several simple equations that are either taken as empirical relationships or derived from various theoretical models.^60^ The most popular is the Langmuir equation describing monolayer adsorption on homogeneous surfaces. The Langmuir model assumes that particles do not change their shape on the surface and ignores the interactions between them. Despite such crude simplifications, this equation is used for hairy particles.^45−48^ Therefore, we approximated the simulated real adsorption isotherm using Langmuir adsorption isotherm
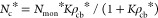
12where *N*_c_^*^ = *N*_c_σ_c_^3^, *N*_mon_^*^ = *N*_mon_σ_c_^3^, and *N*_mon_ is the monolayer capacity (maximal number of cores in the monolayer), while *K* is the adsorption constant involving adsorption energy. These parameters can be calculated by linearization of eq 10.

Figure 14 shows the real adsorption isotherms calculated from the Langmuir equation (lines) and determined from simulations (points). The linear forms of the isotherms are shown in the inset. The following best-fitted parameters were obtained for the considered surfaces: *K* = 2563.77 and *N*_mon_^*^ = 0.2803 (S1), *K* = 5443.96 and *N*_mon_^*^ = 0.2899 (S2), and *K* = 1717.39 and *N*_mon_^*^ = 0.3675 (S3). As expected, the adsorption constant for surface S1 is smaller than for the S2-surface. It is noteworthy, however, that the constant *K* estimated for the surface S3 is smaller than for S2. This is in accord with the relation between the corresponding effective potentials near the walls. The capacity monolayer, *N*_mon_^*^, slightly increases as the parameter ε_s_^*^ increases. Indeed, at first approximation the Langmuir equation can describe the adsorption of hairy particles at low densities quite well. However, the exact interpretation of the obtained constants at the molecular level is difficult or even impossible.

**Figure 15:**
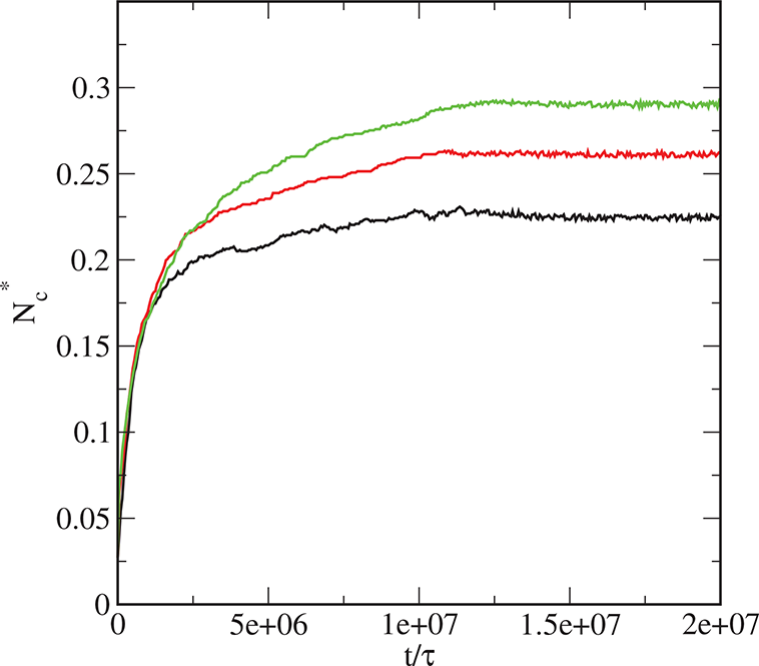
Evolution of the number of adsorbed nanoparticles plotted as a function of time for different surfaces: S1 (black line), S2 (red line), and S3 (green line). The initial density of particles, ρ_*t*0_ = 0.01.

We also analyzed the evolution of the number of adsorbed nanoparticles as a function of time. Examples of such plots are presented in Figure 15. We see here that the adsorption process has reached equilibrium.

In summary, we demonstrated how the strength of chain–substrate interactions affects the adsorption of hairy nanoparticles on solid surfaces. Our research shows that adsorption is a very complicated process. This is due to the competition between interparticle interactions on the substrate and in the bulk phase, and the entropy effects associated with changes of possible chain configurations near the surface.

## Summary

In this work, we present the results of large-scale molecular dynamics simulations of adsorption polymer-tethered particles on solid surfaces. The solvent is involved implicitly. Segment–segment, segment–core, and core–core interactions are purely repulsive, while the segment–substrate interactions are attractive. There is no particle aggregation in the system. First, we analyze the behavior of single hairy particles on the surfaces with increasing strength of the segment–substrate interactions (ε_s_^*^). We calculated the segment density profiles along the axes *x*, *y*, *z*, and the mass dipole moments. We show that the hairy particles change their internal structure at the substrate. As expected, more segments accumulate on more attractive substrates. The segment density near the surface increases, and the core–substrate distance decreases. The shape of the polymer canopy changes in the vicinity of the substrate; on stronger adsorbents the particles become “flattened”. The hairy particles are found to be symmetrical in a plane parallel to the substrate (*xy*) but strongly asymmetric in the direction of *z*. The latter effect is stronger for stronger adsorbents. Our results are consistent with the previous simulation^41^ and experimental studies.^40^

The main part of our results is related to the adsorption of hairy particles dispersed in systems with different particle densities. We consider adsorption on a weakly attractive substrate (S1) and two strong adsorbents (S2, S3). For all systems, we evaluated the local core and segment densities at different initial densities of particles in the system. The shape of the density profiles crucially depends on the strength of segment–substrate interactions. The profiles of the cores have one well-pronounced peak. For the weak adsorbent (S1), this peak is wide and relatively low. By contrast, in the systems with strong adsorbents, these peaks are very narrow and high. Thus, there is monolayer adsorption. The cores are far from the substrate for weak adsorbents and much closer to it for stronger ones. The impact of the density of particles on the position of the cores is not significant. The segment profiles, however, have two peaks near the wall. In the case of the S1-surface, these maxima are low; for the remaining surfaces the first peaks are much higher. At greater distances from the surface S1, the segment density shows a plateau in the region where the cores are located. Then, it continues to decrease smoothly. In the case of the surfaces S2 and S3, plateaus disappeared. It is noteworthy that in the outer part of the monolayer a depletion in the particle density is found in comparison with the bulk phase. In the case of the considered model, we can say that cores rest on the layer of segments.

We have also evaluated the Boltzmann averaged effective particle–substrate potentials. These potentials are softly repulsive in immediate proximity to the wall; then they are attractive in the region of the monolayer of cores and again repulsive at longer distances. The changes of the shape of the function *v*_eff_^*^(z*) with the parameter ε_s_^*^ correspond to the change of the density profiles. For the weakest adsorbent, the attractive well is significantly shallower and wider than those for the stronger adsorbents, while the repulsive part of the effective potential is lower.

For all the considered systems, we found monolayer adsorption of hairy particles. We explored the ordering of the cores at the substrate. In the majority of the systems, the particles are randomly distributed over the surface. However, for the strongest adsorbent and relatively high bulk density, ρ_cb_^*^, the particles form a regular hexagonal array.

We calculated the excess adsorption isotherms for all systems. For relatively high particle densities, the excess adsorption becomes greater as the parameter ε_s_^*^ increases. At low densities a more complicated relation is found. We have also estimated the real adsorption of particles and approximated it using the Langmuir equation. Surprisingly, such an approximation appears to be quite satisfactory. This confirms the possibility of application of this equation to describe experimental results.^45−48^

Our conclusions are qualitatively consistent with the results of experimental studies concerning the morphology of surface layers formed by polymer-tethered particles on different substrates.^40^ The simulations not only reflect the main trends observed for real systems but also give a deeper insight into the structure of surface layers at the “atomistic” level.

In this work, we have focused our attention on the adsorption of hairy particles. To determine the adsorption isotherms, a very large number of particles in the system is needed. We have to generate many systems containing a lot of “atoms”. In order to reduce the computation time, we decided to keep the tethers quite short. However, despite this limitation, our results capture the basic characteristics of hairy particles on surfaces. Moreover, we concentrate on on the role of interactions of the polymer corona with the substrate. Therefore, we consider the simple model involving only attractive segment–substrate interactions. Of course, in real systems other interactions can also be important, in particular, attraction of the cores by the wall and particle–particle attraction resulting in their aggregation.^44^ Such systems are currently under study in our laboratory.

In summary, we have shown how the solid surface influences the behavior of hairy particles, their adsorption, and the structure of adsorbed monolayer films. We hope that our results would provide useful information for future theoretical studies as well as for practical applications in nanotechnology and environmental protection.
